# Maternal Smoking, Alcohol and Recreational Drug Use and the Risk of SIDS Among a US Urban Black Population

**DOI:** 10.3389/fped.2022.809966

**Published:** 2022-05-10

**Authors:** Fern R. Hauck, Sarah R. Blackstone

**Affiliations:** Department of Family Medicine, University of Virginia School of Medicine, Charlottesville, VA, United States

**Keywords:** pregnancy, sudden infant death syndrome (SIDS), smoking, cocaine, alcohol, racial disparities, marijuana, sudden unexpected infant death (SUID)

## Abstract

**Background:**

Rates of sudden infant death syndrome (SIDS) are twice as high among Black infants compared to white infants in the US. While the contribution of sleep environment factors to this disparity is known, little is known about the risk of SIDS among Black infants in relation to maternal prenatal smoking, alcohol and drug use as well as infant smoke exposure.

**Objective:**

To assess the contribution of maternal substance use during pregnancy and the potential interactions with infant bedsharing in a high-risk, urban Black population.

**Methods:**

The Chicago Infant Mortality Study (CIMS) collected data on 195 Black infants who died of SIDS and 195 controls matched on race, age and birthweight. Risk of SIDS was calculated for maternal smoking, alcohol and drug use, adjusting for potential confounding variables and other risk factors for SIDS. Interactions between these substance use variables and bedsharing were also calculated.

**Results:**

Infants were more likely to die from SIDS if the mother smoked during pregnancy (aOR 3.90, 95% CI 1.37–3.30) and post-pregnancy (aOR 2.49, 95% CI 1.49–4.19). There was a dose response seen between amount smoked during pregnancy and risk of SIDS. Use of alcohol (aOR 2.89, 95% CI 1.29–6.99), cocaine (aOR 4.78, 95% CI 2.45–9.82) and marijuana (aOR 2.76, 95% CI 1.28–5.93) were associated with increased risk of SIDS. In the final, multivariable model controlling for sociodemographic factors and covariates, maternal smoking (aOR 3.03, 95% CI 1.03–8.88) and cocaine use (aOR 4.65, 95% CI 1.02–21.3) during pregnancy remained significant. There were significant, positive interactions between bedsharing and maternal smoking during pregnancy and post-pregnancy, alcohol use and cocaine use.

**Conclusion:**

Maternal use of tobacco, alcohol and cocaine during pregnancy is associated with significantly increased risk of SIDS in a Black, urban population. Reducing substance use and eliminating disparities in SIDS, sudden unexpected infant death (SUID) (also known as sudden unexpected death in infancy or SUDI) and infant mortality need to involve more than individual level education, but instead will require a comprehensive examination of the role of social determinants of health as well as a multi-pronged approach to address both maternal and infant health and wellbeing.

## Introduction

Black-white disparities in infant mortality and sudden infant death syndrome (SIDS) in the United States are well known and longstanding. Many would argue that it is a national “tragedy and disgrace” and eliminating these disparities must be a priority (1). Data from 2014 to 2018 show that non-Hispanic Black infants died from SIDS at a rate of 72.7/100,000 live births compared with a rate of 36.7/100,000 live births among non-Hispanic white infants (2). While some locales have been successful in reducing sleep related infant deaths and narrowing the racial disparity, such as the B'More for Healthy Babies initiative in Baltimore (3), other locales have seen not only persistent but widening gaps.

Sudden unexpected infant death (SUID) also known as sudden unexpected death in infancy (SUDI) includes deaths from SIDS, accidental suffocation and strangulation in bed (ASSB) and undetermined causes of death, which previously may have been classified as SIDS (4). In the US, a diagnostic shift occurred between 1999 and 2001, when many medical examiners and coroners began classifying SUID deaths as ASSB or undetermined rather than SIDS (4). Between 1999 and 2015, rates of SIDS in the US decreased by 35% and rates of ASSB increased by 183% (while there were no significant reductions in overall SUID), possibly reflecting a shift in the preferred use of the term ASSB (5). To demonstrate the widening racial/ethnic disparity in SUID, the SUID rate in Illinois for Black infants (3.3/1,000 live births) compared with white infants (0.5/1,000 live births) was almost seven times higher in 2018 (6). While SUID rates among infants born to white and Hispanic mothers have remained stable since 2000 in Illinois, the SUID rate among infants born to Black mothers has increased by 38% since 2009 (7).

Risk factors for SIDS include unsafe sleep circumstances, such as placing infants prone to sleep, with soft bedding, or sharing a sleep surface with other adults or children. The Chicago Infant Mortality Study (CIMS) identified these as significant risk factors in the largely Black population studied (8–10). In addition to infant care practices that increase the risk for SIDS, parental use of tobacco, alcohol or recreational drugs have been shown to also be associated with risk. The relationship between maternal smoking in pregnancy and postpartum passive smoke exposure of infants has been well documented by numerous studies worldwide over the past 3 decades. In a comprehensive review of maternal smoking during pregnancy and risk for SIDS, the pooled relative risk of studies conducted before the Safe to Sleep campaigns (which included recommendations to avoid smoking in pregnancy) was 2.86 (2.77–2.95); the RR of studies conducted after these campaigns was 3.93 (3.78–4.08) (11). It is estimated that up to one-third of SIDS deaths could be prevented if mothers did not smoke during pregnancy. More recent studies continue to show the strong association between prenatal maternal smoking and SIDS as well as a dose effect (12). The New Zealand 2017 SUDI (Sudden Unexpected Death in Infancy) Nationwide Case Control Study found a strong interaction between maternal smoking in pregnancy and bedsharing; infants exposed to both risk factors had a large increased risk of SUDI (SUID) (aOR = 32.8, 95% CI 11.2–95.8) compared with infants not exposed to either risk factor (13). The risk of SIDS is also particularly high when an infant bed shares with an adult smoker, even when the adult is not smoking in bed (OR 2.3–21.6) (10).

The risk of SIDS associated with maternal alcohol and drug use have been less well studied, and results are less consistent. In addition, it is difficult to separate out the effects of individual substances on the risk of SIDS, since multiple substances are often used. Studies of maternal alcohol use during pregnancy have found a six-fold increased risk of SIDS with periconceptional use (aOR 6.2, 1.6–23.3), an eight-fold increased risk with binge drinking in the first trimester (aOR 8.2, 1.9–35.3) (14), a four-fold increased risk with drinking past the first trimester (aOR 3.95, 0.44–35.83) (15) and a seven-fold increased risk when mothers had a diagnosis of alcoholism during pregnancy (adjusted hazard ratio 6.92) (16).

Studies of recreational drug use and SIDS generally focused on individual drugs or drugs in combination. A meta-analysis of 5 studies found that cocaine-exposed infants had a four-fold increased risk of SIDS compared with drug-free infants (unadjusted OR 4.10, 3.17–5.30) (17). Among infants born to maternal drug users, the incidence of SIDS did not differ by race or ethnicity.

To our knowledge, only one published study found a positive association between maternal marijuana use and SIDS. Infants of mothers who used marijuana post-partum had an increased risk of SIDS only when used at night (aOR 2.35, 1.6–4.05), but not during the day (18). As with smoking, parental alcohol and/or recreational drug use in combination with bedsharing places the infant at particularly high risk for SIDS (10).

Very limited research has focused on substance use risk factors for SIDS and SUID within the Black community. The purpose of the current analyses is to examine the risks between maternal smoking, passive smoke exposure, alcohol and drug use and SIDS, based on findings from the Chicago Infant Mortality Study. Additionally, recommendations are made to address the persistent Black-white disparities in SIDS and SUID, with particular attention to social determinants of health (SDOH). SDOH include individual level factors (income, education, marital status, food security, access to healthcare, housing security, experiences with racism and racial discrimination) and community level factors (crime, housing stock, air pollution, toxin exposure, segregation), all of which can affect maternal and child health outcomes (19). Many studies support the potential influence of SDOH on racial/ethnic disparities in preterm birth, but fewer studies have investigated their role in infant deaths (19).

## Methods

### The Chicago Infant Mortality Study

This study involves secondary data analysis from the Chicago Infant Mortality Study (CIMS). The methodology for CIMS is described in detail in previous publications (8, 20, 21). Briefly, CIMS was a population-based, case-control study examining risk factors for SIDS among a largely Black population in Chicago. All Chicago resident infant deaths from November 1993 to April 1996 that were classified as SIDS per the Medical Examiner's office (cases) were matched to living controls based on (in order of priority) maternal race/ethnicity, age at death/interview and birth weight. Data for cases were collected through the death scene investigation which included approximately 400 questions detailing the circumstances before death; the sleep environment of the child when last put down and found; the infant's and family's medical history; the mother's prenatal alcohol, tobacco, and drug use history; and other factors pertinent to determining the cause of death (20). Additionally, standardized autopsy procedures were followed by the Medical Examiners, including gross and microscopic examination, laboratory, toxicology and radiology studies (20). The Medical Examiner determined cause of death based on the autopsy, death scene investigation and review of medical history. One in four cases were reviewed blindly by an external review team, including a forensic pathologist and a pediatric pathologist experienced in sudden infant death. Diagnoses that differed from those of the Medical Examiner or each other were discussed by a multidisciplinary committee to establish the final diagnosis. SIDS was defined as “the sudden death of an infant under 1 year of age, which remains unexplained after a thorough case investigation, including performance of a complete autopsy, examination of the death scene, and review of the clinical history (22).” Two weeks after the death, the infant's caretaker completed a standardized in-home follow-up interview to address items not included in the death scene investigation (e.g., routine infant sleep practices).

Potential control infants meeting matching criteria were identified through review of birth certificates at the Chicago Department of Public Health. Parents of potential controls were sent a letter in the mail inviting their participation. Controls were enrolled on a first-come basis and interviews with their mothers took place in the home. The death scene investigation and follow-up interview questionnaires were reworded to apply to living infants. Control infants' mothers received a small stipend ($25-$35 over the course of the study) for participating.

### Variables

#### Sociodemographic Variables

Key sociodemographic variables from CIMS included maternal age, education, marital status and quality of prenatal care based on the Kessner index (23). Maternal age was categorized as under 25 and 25+ years. Education was grouped into two categories: less than high school and high school diploma or greater. Mothers' marital status was dichotomous: married/non-married. Index of prenatal care was categorized as adequate, intermediate or inadequate. Infants' gestational age in weeks was also included to account for any effect of prematurity. Infant sex was also included.

#### Tobacco, Alcohol and Drug Use

Mothers indicated whether they had smoked during pregnancy and post-pregnancy. Additional measures determined amount smoked during and post-pregnancy, which were grouped as: less than half a pack per day, half a pack to one pack per day and more than one pack per day. Participants also indicated whether they were exposed to other smokers during and post-pregnancy. Alcohol use and binge drinking (≥4 drinks on one occasion) were included. Women also indicated whether they had used cocaine or marijuana during their pregnancy.

#### Covariates

Because of the relationship between sleep environment and SIDS (8, 20), we controlled for use of a soft sleep surface, pillow use, prone sleeping position and bedsharing. All of these variables referred to infants' environment during their last sleep.

### Statistical Analysis

Analyses were conducted using data from the 195 Black SIDS infants and 195 Black control infants. Initially we used the Cochran-Mantel-Haenszel statistic to determine differences in sociodemographic variables, maternal tobacco, alcohol and drug use and additional covariates between the cases and controls. Conditional logistic regression to account for matching was used to obtain unadjusted odds rations (ORs) and corresponding 95% confidence intervals (CIs), and odds ratios adjusted for the four sociodemographic variables (maternal age, education, marital status and Kessner index). A multivariable, conditional logistic regression model was constructed to determine the influence of tobacco, alcohol and drug use while controlling for sociodemographic variables and other covariates. Variables that remained significant after adjusting for sociodemographic factors were included in the final analysis. Due to collinearity between the smoking variables, we only included the measure reflecting maternal smoking during pregnancy.

Interactions were examined between the bedsharing and tobacco, alcohol and drug use variables, as previous literature suggests bedsharing is linked with other risk factors for infant death (13). Variables significant in univariable analyses were included. Significance was determined at *p* < 0.05. All analyses were conducted using R 4.1.1.

## Results

Cases and controls were similar based on matched factors, i.e., mean age for cases and controls (84.9 and 82.1 days, respectively) and birth weight (6.1 pounds and 6.3 pounds) were not significantly different. There were significant differences between cases and controls on non-matched factors. Mean gestational age in cases was 37.4 (SD = 3.1) and 38.3 (SD = 2.4) in controls (*p* < 0.01). Cases were more likely to have mothers with less than high school education, inadequate prenatal care and who were not married. All were significant at *p* < 0.05 (Table 1). Based on previous research (8, 20), maternal age, marital status, education, and adequacy of prenatal care were chosen to represent sociodemographic factors and were therefore used in subsequent analyses for adjustment purposes.

**Table 1a:** Unadjusted and adjusted ORs for demographic and sleep variables.

**Characteristic**	**SIDS cases**	**Controls N = 195^a^**	**OR** **(95% CI)^+^**	**OR** **(95% CI)^±^**
	**N = 195^a^**			
Infant sex				
Female	83/(43%)	97/(50%)	Reference	Reference
Male	112/(57%)	98/(50%)	1.34 (0.89, 1.99)	1.34 (0.87, 2.09)
Maternal age				
25 and over	63/(32%)	78/(40%)	Reference	
Less than 25	132/(68%)	117/(60%)	1.39 (0.92, 2.11)	–
Education				
High school or greater	89/(46%)	139/(71%)	Reference	
Less than high school	106/(54%)	56/(29%)	**2.95** **(1.94, 4.49)**	–
Kessner Index of prenatal care				
Adequate	73/(37%)	113/(58%)	Reference	
Inadequate	70/(36%)	22/(11%)	**4.92** **(2.84, 8.79)**	
Intermediate	52/(27%)	60/(31%)	1.34 (0.83, 2.15)	–
Marital status				
Married	13/(6.7%)	43/(22%)	Reference	
Not married	181/(93%)	152/(78%)	**3.93** **(2.04, 7.59)**	–
Soft sleep surface				
No	94/(48%)	153/(78%)	Reference	Reference
Yes	101/(52%)	42/(22%)	**3.91** **(2.51, 6.09)**	**3.32** **(2.07, 5.36)**
Pillow use				
No	145/(74%)	170/(87%)	Reference	Reference
Yes	50/(26%)	25/(13%)	**2.34** **(1.38, 3.98)**	**2.19** **(1.24, 3.94)**
Prone sleep position				
No	82/(42%)	111/(57%)	Reference	Reference
Yes	113/(58%)	84/(43%)	**1.82** **(1.21, 2.72)**	**1.95** **(1.26, 3.03)**
Bedsharing				
No	82/(42%)	123/(63%)	Reference	Reference
Yes	113/(58%)	72/(37%)	**2.34** **(1.57, 3.53)**	**2.17** **(1.37, 3.30)**

a *n / (%).*

+ *Statistically significant ORs are indicated in bold.*

± *Adjusted for maternal age, marital status, education, and index of prenatal care*.

**Table 1b:** Unadjusted and adjusted ORs for smoking variables.

**Characteristic**	**SIDS cases**	**Controls** **N = 195^a^**	**OR** **(95% CI)^+^**	**OR** **(95% CI)^±^**
	**N = 195^a^**			
Smoked during pregnancy				
No	91/(47%)	151/(77%)	Reference	Reference
Yes	104/(53%)	44/(23%)	**3.92** **(2.53, 6.08)**	**3.90** **(2.35, 6.12)**
Smoked during pregnancy <1/2 ppd^b^				
No	90/(65%)	152/(87%)	Reference	Reference
Yes	49/(35%)	22/(13%)	**3.76** **(1.13, 6.62)**	**3.14** **(1.66, 6.07)**
Smoked during pregnancy ≥1/2 ≤ 1 ppd				
No	90/(73%)	152/(92%)	Reference	Reference
Yes	33/(27%)	13/(8%)	**4.28** **(1.24, 8.57)**	**5.18** **(2.40, 11.89)**
Smoked during pregnancy >1 ppd				
No	90/(83%)	152/(96%)	Reference	Reference
Yes	19/(17%)	7/(4%)	**4.58** **(1.85, 11.33)**	**5.67** **(2.03, 17.55)**
Smoked post-pregnancy (mother)				
No	108/(58%)	153/(78%)	Reference	Reference
Yes	78/(42%)	42/(22%)	**2.63** **(1.67, 4.12)**	**2.49** **(1.49, 4.19)**
Smoked post-pregnancy (mother) <1/2 ppd				
No	108/(71%)	153/(83%)	Reference	Reference
Yes	45/(29%)	31/(17%)	**2.05** **(1.22, 3.46)**	**1.85** **(1.02, 3.39)**
Smoked post-pregnancy (mother) ≥1/2 ≤ 1 ppd				
No	108/(82%)	153/(97%)	Reference	Reference
Yes	24/(18%)	5/(3%)	**6.80** **(2.51, 18.38)**	**7.78** **(2.86, 25.31)**
Smoked post-pregnancy (mother) >1 ppd				
No	108/(92%)	153/(96%)	Reference	Reference
Yes	9/(8%)	6/(4%)	2.12 (0.73, 6.14)	1.92 (0.62, 6.32)
Exposed to other smokers while pregnant (mother)				
No	76/(39%)	107/(55%)	Reference	Reference
Yes	119/(61%)	88/(45%)	**1.90** **(1.27, 2.84)**	**4.00** **(2.55, 6.36)**
Exposed to other smokers post-pregnancy (mother)				
No	70/(36%)	142/(73%)	Reference	Reference
Yes	125/(64%)	53/(27%)	**4.78** **(3.11, 7.35)**	1.43 (0.92, 1.23)
Number of smokers exposed to during pregnancy (mother)				
None	57/(29%)	124/(65%)	Reference	Reference
One	71/(36%)	43/(23%)	**3.59** **(2.21, 5.91)**	**3.59** **(2.21,5.91)**
More than one	67/(34%)	23/(12%)	**6.33** **(3.64, 11.37)**	**6.34** **(3.64, 11.37)**

a *n/(%).*

b *ppd, packs per day.*

+ *Statistically significant ORs are indicated in bold.*

± *Adjusted for maternal age, marital status, education, and index of prenatal care*.

**Table 1c:** Unadjusted and adjusted ORs for alcohol and drug variables.

**Characteristic**	**SIDS cases**	**Controls** **N = 195^a^**	**OR** **(95% CI)^+^**	**OR** **(95% CI)^±^**
	**N = 195^a^**			
Drank alcohol during pregnancy				
No	138/(82%)	156/(95%)	Reference	Reference
Yes	31/(18%)	9/(5%)	**3.89** **(1.79, 8.46)**	**2.89** **(1.29, 6.99)**
Binge drinking during pregnancy				
No	136/(92%)	190/(98%)	Reference	Reference
Yes	12/(8%)	4/(2%)	**4.19** **(1.32, 13.27)**	2.86 (0.91, 10.99)
Cocaine use during pregnancy				
No	133/(68%)	180/(92%)	Reference	Reference
Yes	62/(32%)	15/(8%)	**5.59** **(3.04, 10.26)**	**4.78** **(2.45, 9.82)**
Marijuana use during pregnancy				
No	159/(82%)	184/(94%)	Reference	Reference
Yes	36/(18%)	11/(56%)	**3.79** **(1.87, 7.67)**	**2.76** **(1.28, 5.93)**

a *n/(%).*

+ *Statistically significant ORs are indicated in bold.*

± *Adjusted for maternal age, marital status, education, and index of prenatal care*.

There were several differences in tobacco, alcohol and drug use between cases and controls after controlling for sociodemographic variables. Infants were more likely to die from SIDS if the mother smoked during pregnancy (aOR 3.90, 95% CI 2.35, 6.12) and post-pregnancy (aOR 2.49, 95% CI 1.49–4.19). There was a dose response seen between amount smoked during pregnancy and risk of SIDS, with the odds ratio increasing from 3.14 (95% CI 1.66–6.07) for smokers of less than a half pack of cigarettes per day to 5.67 (95% CI 2.03–17.55) for smokers of more than 1 pack per day. This was also seen in smoking post-pregnancy, as the odds ratio for smokers of less than half a pack of cigarettes per day (aOR 1.85, 95% CI 1.02–3.39) increased for smokers of half a pack to one pack of cigarettes per day (aOR 7.78, 95% CI 2.86–25.31). Exposure to other smokers while pregnant (aOR 4.00, 95% CI 2.55–6.36) was associated with increased risk of SIDS, while maternal exposure to other smokers post-pregnancy was not significant (aOR 1.43, 95% CI 0.92–1.23). The risk of SIDS increased as the number of smokers pregnant women were exposed to increased (for more than one other smoker, aOR 6.34, 95% CI 3.64–11.37). Use of alcohol (aOR 2.89, 95% CI 1.29–6.99), cocaine (aOR 4.78, 95% CI 2.45–9.82) and marijuana (aOR 2.76, 95% CI 1.28–5.93) during pregnancy were associated with increased risk of SIDS.

In the final, multivariable model controlling for sociodemographic factors, gestational age, and covariates, maternal smoking (aOR 6.15, 95% CI 1.55, 24.4) and cocaine use (aOR 8.91, 95% CI 1.05, 75.6) during pregnancy remained significant (Table 2). Maternal alcohol and marijuana use during pregnancy were no longer significant. Sleep environment variables were significantly associated with SIDS. During last sleep, use of a soft sleep surface (aOR 9.01, 95% CI 2.83, 28.7), pillow use (aOR 6.74, 95% CI 1.39, 32.6), prone sleeping position (aOR 3.72, 95% CI 1.37, 10.1) and bedsharing (aOR 3.30 95% CI 1.04, 10.4) were associated with infant death.

**Table 2:** Multivariable analysis of risk factors for SIDS.

**Characteristic**	**OR^a^**	**95% CI^a^**	***p* value**
Maternal age			
25 and over	Reference		
Less than 25	1.44	0.50, 4.14	0.5
Education			
High school or greater	Reference		
Less than high school	4.72	1.48, 15.1	0.009
Kessner Index of prenatal care			
Adequate	Reference		
Inadequate	2.15	0.65, 7.06	0.2
Intermediate	1.22	0.35, 4.16	0.8
Mother's marital status			
Married	Reference		
Not married	1.70	0.38, 7.68	0.5
Soft sleep surface			
No	Reference		
Yes	9.01	2.83, 28.7	<0.001
Pillow Use			
No	Reference		
Yes	6.74	1.39, 32.6	0.018
Prone sleeping position			
No	Reference		
Yes	3.72	1.37, 10.1	0.010
Bedsharing			
No	Reference		
Yes	3.30	1.04, 10.4	0.042
Smoked during pregnancy			
No	Reference		
Yes	6.15	1.55, 24.4	0.010
Cocaine use during pregnancy			
No	Reference		
Yes	8.91	1.05, 75.6	0.045
Marijuana use during pregnancy			
No	Reference		
Yes	1.74	0.29, 10.6	0.5
Drank alcohol during pregnancy			
No	Reference		
Yes	0.44	0.07, 2.99	0.4

a *OR, odds ratio; CI, confidence interval.*

*All variables in the multivariable model are shown in the table*.

There was a significant, positive interaction between bedsharing and smoking during pregnancy indicating the combined presence of both factors had a greater effect than would be expected by simply multiplying the effects alone. After adjusting for the four confounding variables, the OR was 8.4 (95% CI 4.06–17.38). There were also significant, positive interactions between bedsharing and amount smoked. The adjusted odds ratio for smoking half a pack to one pack of cigarettes per day was 8.56 (95% CI 2.93–24.97) and increased to 25.73 (95% CI 4.67–41.77) for smokers of greater than one pack per day. Additionally, there was a significant, positive interaction between smoking post-pregnancy and bedsharing (aOR 4.72, 95% CI 2.35–9.46). Smoking half to one pack of cigarettes per day post-pregnancy demonstrated a significant interaction with bedsharing (aOR 14.44, 95% CI 3.74–55.79). There was a significant positive interaction between bedsharing and exposure to other smokers post-pregnancy (aOR 7.58, 95%, 3.92–14.65), alcohol use (aOR 6.39, 95% CI 2.01–20.37) and cocaine use (aOR 14.43, 95% CI 4.62–45.04).

## Discussion

In our study of 195 Black infants who died from SIDS and 195 matched controls, we found that maternal smoking during pregnancy, maternal passive smoke exposure during pregnancy, and post-partum infant smoke exposure were all strongly associated with an increased risk of SIDS, controlling for socio-demographic variables. In the final multivariable model, we included only maternal smoking in pregnancy, since the smoking variables are highly correlated with each other. Smoking remained a significant risk factor. This is consistent with studies conducted before and after campaigns were launched in countries around the world to emphasize back sleeping and other practices to reduce the risk of SIDS. CIMS was conducted during the period (1993–1996) when non-prone sleep recommendations were being made on a national basis. The American Academy of Pediatrics advised against prone sleeping in 1992, endorsed by the national Back to Sleep Campaign (now called Safe to Sleep) in 1994, revised in 1996 to recommend back sleeping only. While most attention was focused on infant sleep position, the campaigns generally also included advice to avoid smoking during pregnancy and exposure of the infant to environmental smoke. Reductions in SIDS rates as a result of campaigns have been largely credited to reductions in prone sleeping rates, but there is also evidence that campaigns have resulted in reductions in maternal smoking in pregnancy (24).

In the US, rates of maternal smoking during pregnancy have declined among all racial/ethnic groups over the period 2010–2017. Black pregnant women smoke at lower rates than white and American Indian/Alaska Native women (25). However, women with lower education are more likely to smoke within racial/ethnic groups. Healthy People 2030 provides data driven national objectives to improve the health and well-being of American people over the next decade (26). The target for smoking abstinence during pregnancy is 95.7%; in 2019 it was 94%. Thus as a nation, the target is within reach. However, compared to white smokers, Black smokers had significantly lower odds of being asked about tobacco use by healthcare providers (aOR 0.70), being advised to quit (aOR 0.72), or having used tobacco-cessation aids during the past year in a quit attempt (aOR 0.60), after controlling for sociodemographic and healthcare factors (27). Research has shown that advice from a health professional to quit smoking does motivate people to quit, thus these lower rates of counseling about smoking cessation can impact smoking among Black pregnant women (28).

We found an almost three times increased risk for SIDS when mothers drank alcohol or binge drank alcohol during pregnancy, controlling for sociodemographic factors. This became non-significant in the larger multivariable model. These results are consistent with other studies where the results were less convincing of a relationship between maternal alcohol use during pregnancy and SIDS (16, 29, 30). Since alcohol use is highly correlated with smoking, alcohol use appears to be contributing less to SIDS risk. In the Safe Passage Study, there was a higher risk of alcohol use combined with smoking beyond the first trimester than either alone (15).

Generally, pregnant women consume less alcohol than non-pregnant women, but racial/ethnic differences have been found regarding drinking behavior during pregnancy. Black women are less likely to reduce alcohol consumption or binge drinking during pregnancy than white women (31). We were unable to find any research investigating if there are differences in counseling about alcohol use during pregnancy by race/ethnicity, but unplanned pregnancies are higher among Black women (32), and unintended pregnancy is associated with later entry into care and higher rates of alcohol and drug use (33). Individual- and neighborhood-level economic disadvantage was shown to predict lower alcohol treatment completion for Blacks and in urban areas in the US, there is a higher density of alcohol outlets in Black and other minority neighborhoods compared with white neighborhoods (34).

Finally, our data show that maternal cocaine and marijuana use during pregnancy increased the risk of SIDS three- to five-fold, respectively, after adjusting for sociodemographic variables. Cocaine use remained significant in the multivariable model while marijuana use became non-significant. Findings from other studies of cocaine use and SIDS are inconsistent, although most did report a positive association similar to ours (17). Cocaine use was very high among CIMS case mothers, and more prevalent that alcohol or marijuana use in this sample. A report from the National Drug Intelligence Center described the very active drug trade in Cook County, Illinois (where Chicago is located), and heroin and cocaine were estimated to be used by about 6% of the population, 80% of which was cocaine and 12% was cocaine plus heroin (1995 data) (35). Thus, cocaine was very available at the time of the study. Unfortunately, more recent reports indicate that drug activity has not abated in Chicago, and that cocaine and other illicit drugs are still very much available (36). It is therefore essential that this threat to maternal and infant health be given the necessary attention regarding assessment and treatment.

The weight of evidence to date indicates that marijuana use is not associated with increased risk of SIDS. However, this may be a reflection of a number of other factors such as inadequate power to detect an effect and the correlation of marijuana use with other substances including tobacco. It is estimated that about 8% of women in the US reported using marijuana in the past month and 4% of pregnant women reported use in the past month (37). As more states have liberalized use of recreational and medicinal marijuana, there is growing perception of its safety. At the same time the concentration of delta-9-tetrahydrocannabinol, the active ingredient in marijuana, has increased and this chemical crosses the placenta. The research on adverse maternal and child health outcomes has had mixed results but there is evidence that maternal marijuana use can cause increased neonatal infectious or neurologic morbidity and developmental and behavioral difficulties in children. Based on these potential adverse outcomes, it is recommended that women be screened for marijuana use when planning for or early in pregnancy, and be advised to quit.

We found significant interactions between smoking in pregnancy and bedsharing in risk for SIDS. Adjusting for sociodemographic factors, the OR was 8.40 (95% CI 4.06–17.38) and increased to 25.73 (95% CI 4.67–41.77) for mothers smoking more than one pack per day. While not as high as the odds ratio found for this interaction in the New Zealand SUDI Nationwide Case Control Study, the investigators also reported a very increased risk of SUID (SUDI) among infants exposed to both maternal smoking in pregnancy and bedsharing (aOR = 32.8, 95% CI 11.2–95.8) (15). We also found an interaction for maternal smoking post pregnancy and bedsharing, as well as a dose effect. Additionally, there were significant positive interactions between bedsharing and maternal pregnancy alcohol use and cocaine use; to our knowledge this is the first report of these associations. Women who plan to bedshare or who are bedsharing with their infants need to be advised of the high increase in risk for their infants if they are also smoking (or if their infants are exposed to other smokers), or were using alcohol or cocaine during pregnancy. The approach to counseling and motivating changes in these behaviors is extremely challenging and requires inputs on several levels. New paradigms for education and outreach among high risk Black families are very much needed to assist with smoking, alcohol and drug use cessation. This is discussed in greater detail under Addressing Social Determinants of Health to Overcome Racial Disparities below.

### Limitations

As with all studies of SIDS, our study was necessarily retrospective and constrained in size by the relative rarity of the outcome. Recall bias may occur if mothers of SIDS infants recall exposures more thoroughly than mothers of unaffected, healthy infants, thus resulting in an apparent association when there is none. However, prospectively collected data on sleep position have confirmed results from previous retrospective studies, indicating that recall bias has not been a major problem in case-control studies of SIDS (38, 39). It has been shown that the length of time lapsed between the exposure and the recall has a greater influence on recall accuracy (39). In this study, parents of both SIDS victims and control infants were interviewed about their infant's sleep position within a short time of the sleep period. Thus, we do not believe that recall bias was a problem in this study.

Although the CIMS dataset is the largest comprehensive case-control dataset of SIDS in the United States and with the largest number of Black participants, over 2 decades have passed since the study's completion. Nevertheless, we believe that our results remain relevant due to the similarity in prevalence of several of the risk factors, such as bedsharing and soft bedding, to current US prevalences, as well as the similarity in many of our results (10). A study analyzing 4,929 infants in the Centers for Disease Control and Prevention's SUID case registry who died 2011–2017 (37% of whom were Black infants) found that for the 1,548 cases classified as explained, suffocation or unexplained, possible suffocation, 74% were attributed to soft bedding, 20% to overlay, 7% to wedging, and 5% to other (40). The CDC analyzed 2015 data on non-supine sleep position, soft bedding and bedsharing at 6 weeks postpartum from the Pregnancy Risk Assessment Monitoring System (PRAMS) (41). PRAMS is a state-specific and population-based surveillance system that monitors self-reported behaviors and experiences before, during, and shortly after pregnancy among women with a recent live birth. Overall, 20% of mothers reported placing their infants non-prone for sleep; Black infants had the highest prevalence of all racial/ethnic groups (38% compared with 16% of white infants). Almost two-thirds (61%) of respondents reported bedsharing with their infant and 39% of respondents reported using soft bedding in the sleep environment. Bedsharing was more common among Black infants (77%) compared with white infants (53%), as was use of soft bedding (41 vs. 33%, respectively). It is therefore likely that our results are still relevant to Black communities despite the amount of time that has elapsed since data collection.

CIMS cannot directly address the reasons for the Black-white disparities in SIDS, as the focus of the study was to determine if the risk factors identified in other studies, largely conducted outside of the US in non-Black populations, were similar for American Black infants; the methodology thus included matching cases to controls on race/ethnicity. In our first publication of CIMS results, we looked at the risk of prone positioning at last sleep comparing Black with all other infants (20). Because the prone prevalence was high among all cases but low for white control infants compared with Black control infants, the odds ratios for prone sleeping were higher for the white infants. However, when taking into account the overall higher rates of prone sleeping among Black infants, the population attributable risk for this factor was found to be 19% compared to 12% for the other infants. Over time, as noted above, the gap in prone positioning has widened greatly between Black and non-Black infants, as have the differences in bedsharing. Thus sleep position and bedsharing, in conjunction with smoking and substance use, likely explains at least in part the persistent racial/ethnic disparities in SIDS and SUID rates. New case-control studies are needed to identify which risk factors may vary according to race/ethnicity to help further understand these persistent disparities.

Another limitation is that the small cell sizes for substance use limit the precision of the estimates, resulting in wide confidence intervals. Thus, the data should be interpreted with caution.

### Interventions to Address Maternal Smoking, and Alcohol and Drug Use in Pregnancy

An in-depth discussion of interventions to assist women who are contemplating pregnancy or who are pregnant in stopping the use of tobacco, alcohol and recreational drugs is beyond the scope of this paper, however, we would like to provide a few basic principles; a summary of several interventions can also be found in a paper by Hauck and Tanabe (42). A systematic review found that a medical professional's advice to quit smoking modestly increases the quit rate over no advice (28). This would require that obstetrical clinicians inquire about tobacco use (in all its forms including e-cigarettes and vaping) in pregnancy routinely and systematically spend a few minutes asking about the patient's motivation to quit and counseling her on the benefits of quitting for both her health and the health of her infant. Provision of resources such as contact information for smoking cessation programs/quit lines via phone or internet and follow-up are advised. The American College of Obstetrics and Gynecology Committee on Obstetric Practice recommends consideration of pharmacologic intervention in addition to behavioral and psychosocial intervention (43). Other interventions that have been used include health education, feedback, financial incentives and support from friends or partners (44).

There is limited evidence to identify programs that are effective in aiding pregnant women to stop using alcohol. A randomized controlled trial comparing brief counseling by the obstetrical provider compared with 6 intensive nurse-provided education sessions resulted in similar reductions in alcohol and drug use during pregnancy (45). A computer-tailored intervention was found to be more effective in reducing alcohol use compared with midwife delivered counseling or routine care (46). State policies, such as mandatory warning signs, priority treatment for substance abuse programs and prohibitions on criminal prosecution have not been found to be effective (47).

Few studies have identified successful treatment approaches for women using cocaine in pregnancy. One, utilizing contingency management therapy (positive reinforcement with monetary vouchers), was more successful in reducing cocaine use compared with a community reinforcement approach (48). Interventions focused on marijuana use in pregnancy have not been studied to our knowledge, but motivational interviewing, cognitive behavioral therapy and contingency management have been found to be effective in women using marijuana in general (49).

We were unable to identify studies of any smoking, alcohol or drug cessation programs that specifically target Black women. On the contrary, research has shown that Black communities have been victims of corporate activity, “through producing and promoting products harmful to health. The tobacco industry's disease-promoting activities are among the most powerful corporate influences on inner city health. Such activities have included targeted marketing, thwarting and undermining tobacco control efforts, deceptive scientific practices, and influencing policymakers and community leadership groups (50).” Among these corporate activities during the past several decades, the tobacco industry targeted inner cities populated predominantly by low-income Black residents with highly concentrated menthol cigarette marketing (50). A study of advertisements for tobacco and alcohol products in a sample of 24 Black and 11 general audience newspapers in 24 cities identified a greater number of alcohol product advertisements in the Black newspapers and less alcohol and tobacco control advertising (51).

### Addressing Social Determinants of Health to Overcome Racial Disparities

It is apparent that overcoming racial disparities in SIDS/SUID/SUDI and infant mortality requires a multi-pronged approach that digs deeply into understanding the social determinants of health underlying these disparities. Taylor and coauthors describe comprehensive policy recommendations to eliminate racial disparities in maternal and infant mortality, based on the theory of targeted universalism—“an equity framework that employs targeted strategies to achieve a universal goal, namely meeting the needs of all populations, but having an intentional focus on those most in need—Black women and families (1).”

These recommendations target five areas: (1) improve access to critical services by strengthening existing health programs and supporting reproductive health care, screening and treating women at risk for preterm birth, eliminating maternity care deserts, and offering Black women tools to navigate the health care system; (2) improve the quality of care provided to pregnant women by training providers to address racism and build a more diverse health care workforce, creating standardized assessments for mothers and infants, and adopting new models of care and link payment to quality; (3) address maternal and infant mental health by identifying barriers to accessing maternal mental health services, dismantling care barriers with a comprehensive approach and screening for and addressing infant and early childhood mental health issues; (4) enhance supports for families before and after birth by investing in and expanding access to policies and programs that support families' basic needs, investing in community programs that offer one-stop comprehensive services, simplifying enrollment across public benefit programs, investing in home visiting and funding community-based education and communications initiatives to support families; and (5) improve data collection and oversight by standardizing birth and death certificate data, mandating and funding fetal and infant mortality review committees, and ensuring equity in the review process (1). We would propose that this framework be used in all countries and locales where racial/ethnic disparities in SIDS and SUID/SUDI exist.

## Conclusions

We found that maternal use of tobacco, alcohol and cocaine during pregnancy is associated with significantly increased risk of SIDS in a Black, urban population. Reducing substance use and eliminating disparities in SIDS, SUID and infant mortality need to involve more than individual level education, but instead will require a comprehensive examination of the role of social determinants of health, as well as development of a multi-pronged approach to address both maternal and infant health and wellbeing.

## Data Availability Statement

The original contributions presented in the study are included in the article/supplementary materials; further inquiries can be directed to the corresponding author/s.

## Ethics Statement

The studies involving human participants were reviewed and approved by the Institutional Review Board for Health Sciences Research, University of Virginia. The patients/participants provided their written informed consent to participate in this study.

## Author Contributions

FH developed the study idea and drafted the first version of the manuscript. SB conducted the data analysis and drafted the methods and results portions of the manuscript. Both authors reviewed and edited the final version of the manuscript.

## Funding

This work was supported by the National Institute of Child Health and Human Development and the National Institute on Deafness and Other Communication Disorders under Contract Number NO1-HD-3-3188 and the Centers for Disease Control and Prevention and the Association of Teachers of Preventive Medicine under Cooperative Agreement Number U50/CCU300860-06.

## Conflict of Interest

The authors declare that the research was conducted in the absence of any commercial or financial relationships that could be construed as a potential conflict of interest.

## Publisher's Note

All claims expressed in this article are solely those of the authors and do not necessarily represent those of their affiliated organizations, or those of the publisher, the editors and the reviewers. Any product that may be evaluated in this article, or claim that may be made by its manufacturer, is not guaranteed or endorsed by the publisher.
